# Immune Response at the Crossroads of Atherosclerosis and Alzheimer's Disease

**DOI:** 10.3389/fcvm.2022.870144

**Published:** 2022-07-06

**Authors:** Natalie Stahr, Elena V. Galkina

**Affiliations:** Department of Microbiology and Molecular Cell Biology, Eastern Virginia Medical School, Norfolk, VA, United States

**Keywords:** atherosclerosis, Alzheimer's disease, immune response, inflammation, aging

## Abstract

Alzheimer's disease (AD) and cardiovascular disease (CVD) are pathologies that are characterized by common signatures of vascular dysfunction and chronic inflammation that are accelerated with aging. Importantly, epidemiological studies report an independent interaction between AD and CVD and data suggest that chronic inflammation in CVD may accelerate AD development. Atherosclerosis affects most large to medium sized arteries including those supplying the cerebral circulation. Vascular dysfunction caused by atherosclerosis results in blood brain barrier breakdown, inflammation, an impaired clearance of amyloid-beta (Aβ), and finally ends with neurovascular dysfunction. Numerous data indicate that innate and adaptive immune responses shape atherogenesis and increasing evidence suggests an implication of the immune response in AD progression. Currently, mechanisms by which these two diseases are interconnected with each other are not well-defined. In this review, we discuss the recent advances in our understanding of the intertwined role of the immune response in atherosclerosis and AD and the implications of these findings for human health.

## Introduction

Atherosclerosis is a multi-factorial disease of the large to medium arteries, characterized by the buildup of plaque and aggregation of immune cells in response to subsequent endothelial damage (1–4). Alzheimer's Disease (AD) is a neurodegenerative disease characterized by deposition of amyloid β (Aβ) and hyperphosphorylated tau in the brain, leading to significant cognitive decline (5–7). Although these diseases may seem entirely disparate at first glance, recent research has indicated that both share commonalities in their activation and progression (8, 9). Atherosclerosis and AD are both aging-related diseases that can be described as having common signatures of chronic, low-grade inflammation. Recent epidemiological data, explored in more detail herein, supports the notion that these diseases are intertwined. Importantly, these diseases are both heavily modulated by the immune system, with new insights into AD pointing toward a role for peripheral immune dysfunction in its etiology (4, 6, 10). Neuroinflammation in isolation has been explored extensively, but researchers are only beginning to uncover how the peripheral immune system, and its dysfunction during atherosclerosis, may affect the development of AD. Atherosclerosis and AD share many features in immune cell activation and cytokine release that could change how we view AD as a disease. The effect of hyperlipidemia on immune cell subset differentiation and activation in atherosclerosis may additionally alter or exacerbate the pathology of AD (11). Although a multitude of features connect AD and atherosclerosis, including oxidative distress, mitochondrial dysfunction, hypertension, endothelial dysfunction, vascular aging, the gut-brain axis and hyperlipidemia, this review will particularly focus on the interconnections between peripheral immune cell subsets and their dysfunction in atherosclerosis and AD. This review will therefore follow commonalities in the roles of different immune cell subsets in these two diseases, identifying possible links that need further exploration.

## Atherosclerosis and Associated Immune Response

Atherosclerosis is a complex, inflammatory disease associated with lipid accumulation, vascular dysfunction, and inflammatory immune response in the large and medium sized arteries (1–4). Importantly, atherosclerosis continues to be the leading cause of CVD (1–4). Atherosclerosis affects most large to medium sized arteries, including those supplying the cerebral circulation (12). Vascular dysfunction caused by atherosclerosis results in small vessel disease, a breakdown of the blood brain barrier, inflammation, an impaired clearance of Aβ, and finally in neurovascular dysfunctions (13). Many elements that increase the risk of CVD including aging, hypertension, hyperlipidemia, inflammation, and vascular dysfunction also elevate the chances of getting AD (14, 15). Chronic inflammation in the aorta supports atherogenesis, and both innate and adaptive immune responses are involved in this process. The pathological role of neutrophils, Th1, T follicular helper (Tfh), NK and NKT cells, and the protective role of Tregs is established in atherosclerosis, whereas the role of Th2 and Th17 cells remains under debate (1, 3, 4). The role of B cells in atherosclerosis is complex and B cell-subset-specific (16). Follicular (FO) B cells and innate response activator (IRA) B cells are pro-atherogenic, supporting the Th1 response and the production of pro-inflammatory cytokines and immunoglobulins (Igs) (16). Conversely, protective B1a and B1b cells secrete natural antibodies (Abs) that attenuate oxLDL uptake by macrophages (MΦs). A recent study highlighted an anti-atherogenic role of marginal zone B cells (MZ) *via* the regulation of Tfh cell development (16). The role of regulatory B cells remains controversial. Overall, the immune system is strongly involved in atherogenesis and generates a unique, complex immune response that might affect development of other atherosclerosis-associated pathologies. In this review, we provide evidence for an implication of atherosclerosis in the promotion of AD and discuss potential mechanisms by which a unique immune response developed during atherogenesis might affect progression of AD.

## Alzheimer's Disease and Associated Immune Response

Alzheimer's Disease (AD) is a progressive neurodegenerative disorder characterized by the buildup of amyloid β (Aβ) in the brain, followed by neuronal death, and culminates in significant cognitive decline (17, 18). Although numerous etiological theories exist for AD, the predominant hypothesis, the Amyloid Hypothesis, posits that the accumulation of Aβ itself acts as the initial trigger for neurodegeneration (6, 19). Normally, Aβ is cleared by multiple mechanisms including flow–dependent clearance of the interstitial fluid into the cerebral spinal fluid, Aβ uptake by microglia and astrocytes, transport of Aβ by low-density lipoprotein receptor-related protein 1, very low-density lipoprotein receptor and P-glycoprotein, and *via* clearance by proteases such as neprilysin, insulin-degrading enzymes, matrix metalloprotease-9 (MMP9), and glutamate carboxypeptidase II (20). There then emerges different mechanisms that can lead to the accumulation of Aβ in the brain: excessive production of Aβ and decreased clearance of Aβ from the brain.

Early onset AD, also known as familial AD, is caused by one or more mutations in the genes of APP or presenilin1/2, subunits of the γ-secretase enzyme, leading to AD symptoms as early as 30–50 years old with homozygous dominant inheritance (7). These mutations cause an increase in the amyloidogenic pathway, resulting in higher production of Aβ. The more prevalent form of AD, late onset AD, also known as sporadic AD, appears to be caused by the intermingling of genetic and environmental risk factors, which may play a role in either Aβ overproduction and reduction in clearance. Multiple genetic factors feeding into AD have been identified, including isoforms of the Apolipoprotein E (*APOE*) gene and heterozygous mutations in triggering receptor expressed on myeloid cells 2 (TREM2), among many others (7). Of note, mutations in *APOE* and *TREM-2* are also strongly associated with atherogenesis *via* dysregulation of macrophage autophagy making these targets promising candidates for therapeutic manipulation for both pathologies. Although genetics do appear to play a major role in late onset AD, with some estimating a 70:30 split between genetics and environment in the etiology of the disease (21), multiple vascular risk factors are associated with increased risk for dementia such as smoking, diabetes, hypertension, and atherosclerosis (22).

While the role of Aβ in AD is well-established, increasing evidence also suggests an impact of the immune response and associated inflammation in the induction and persistence of AD pathologies (6). It was known for a long time that AD is strongly associated with increased levels of CRP and pro-inflammatory cytokines such as IL-1β, IL-6, and TNFα and some but not all studies found a correlation between levels of peripheral inflammation, aging (23) and AD in humans (24–28). Additionally, several studies demonstrate that elevated levels of peripheral inflammation in midlife to late life correlates with higher risk of dementia and is associated with future cognitive decline and vascular risks (29, 30). Importantly, elevated TNFα levels and reduced TGFβ production have been detected in the cerebrospinal fluid (CSF) of patients with mild cognitive impairment highlighting that CNS-associated inflammation is an early hallmark in the pathogenesis of AD (31). Increased levels of TNFα are also associated with a 2-fold increase in the rate of cognitive decline over a 6-month period (32). Additional evidence on the impact of inflammation are coming from studies demonstrating systemic inflammation induced by either common acute or chronic infections or critical illness might result in AD-like neuropathology in mice (33) and cognitive decline in aged patients (6, 34, 35). These reports imply that systemic inflammation can precede AD-like pathology and likely participate in its induction/acceleration. To date, several lines of evidence including preclinical and genetic data as well as animal studies support the notion that activated immunity accompanies AD pathology at all stages of disease development (9, 36). The pathological role of infiltrating neutrophils and monocytes has been documented in animal models of AD, and a dominant response of Th1 and Th17 cells is a hallmark of the immune response in AD, while a role of Tregs and B cells is still controversial (6, 10). It remains to be determined how peripheral immunity might affect the activation of AD-specific degenerative processes and it is unclear at which stage(s) of AD progression the immune system and associated inflammation play a critical role.

## Epidemiological Perspective of the Atherosclerosis and AD Interconnection

The main risk factor for AD and atherosclerosis is advanced age (5). Aging is accompanied by systemic changes to the innate and adaptive immune systems characterized by chronic inflammation and senescence for vascular and immune cell types. The underlying mechanisms leading to AD have yet to be fully elucidated, but epidemiological data point to connections between aging, AD and atherosclerosis mediated by hyperlipidemia, hypometabolism, hypertension, obesity, and atherosclerotic plaque burden (37). It has long been known that cholesterol and lipid metabolism play a critical role in the development of AD, as seen with the APOE4 isoform of APOE, which increases plasma low-density lipoprotein levels (thereby also increasing risk of atherosclerosis), while also increasing the risk for AD (11). Xiang et al. reported that higher carotid intermedial thickness, as a measure of carotid atherosclerosis, is associated with faster cognitive decline (38). Interestingly, post-mortem studies link intracranial atherosclerosis to increased risk for dementia independent of cerebral infarcts suggesting potentially additional pathways unrelated to AD through which vascular changes may influence dementia risk (39). Cortez-Canteli et al. found that carotid plaque burden was associated with increased risk of hypometabolism independent of CVD risk in middle-aged asymptomatic individuals (40). These important data reveal the role of unmitigated cardiovascular risk factors and subclinical atherosclerosis in the establishment of AD-like symptoms in a relatively young group of subjects. Further strong arguments for an implication of vascular risk factors into AD progression came from a study demonstrating that AD patients that were treated for their vascular risk factors had reduced cognitive decline compared to those that did not receive treatment, despite exclusion of patients with pre-existing cardiovascular disease (41). While epidemiological data strongly suggest an impact of atherosclerosis on AD, there is a limited number of *in vivo* studies, which recapitulate conditions of aging, atherosclerosis and AD. To fill out this gap, development of new models that will display characteristics of both pathologies is necessary as they might provide an important basis for our understanding mechanisms of atherosclerosis-associated AD development and lead to a successful creating an effective and complex pharmacotherapy for patients with AD and atherosclerosis.

## Neutrophils Play a Pathological Role in AD and Atherosclerosis

**Figure 1:**
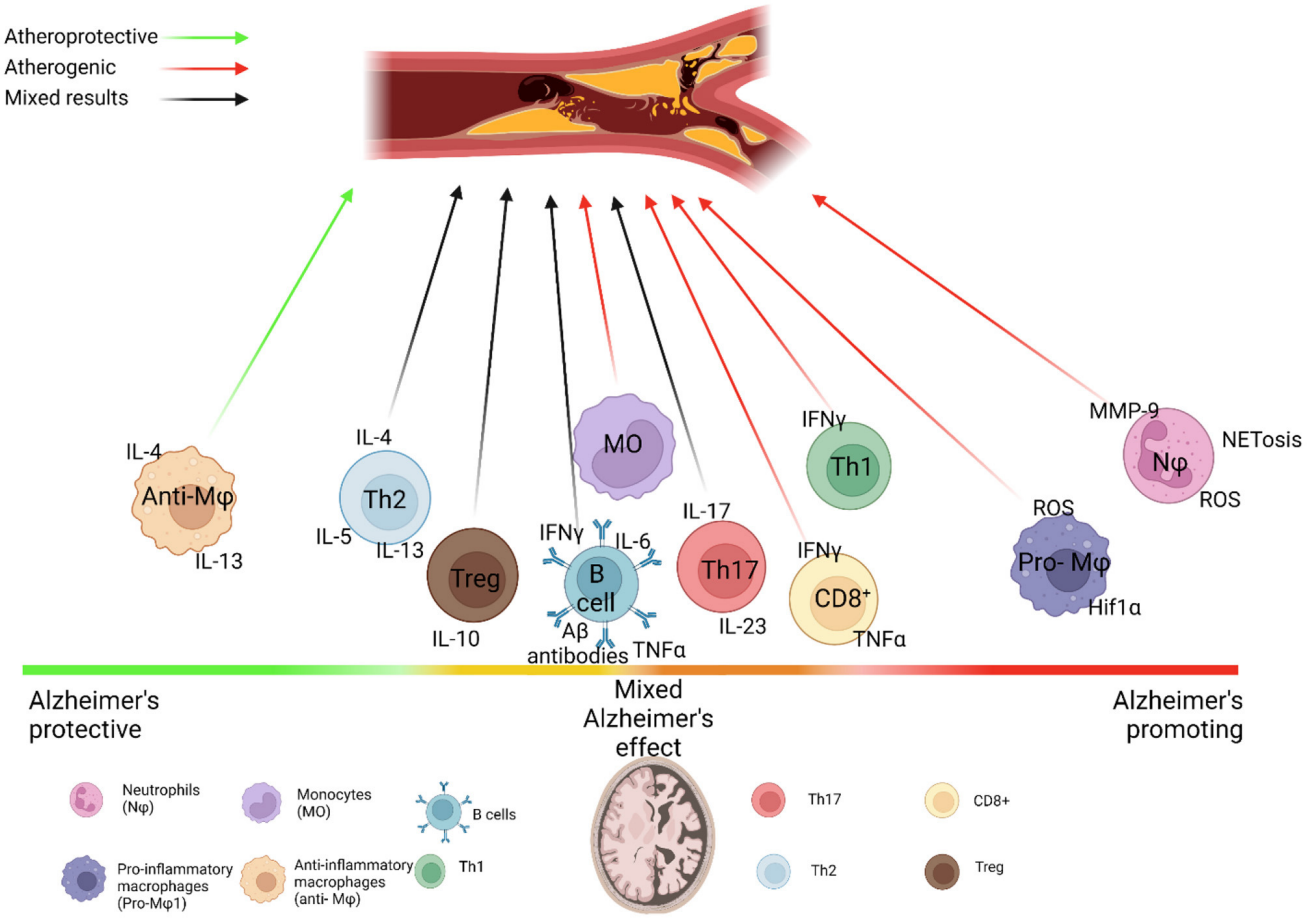
Immune system dysfunction links Alzheimer's and Atherosclerosis. Immune cell subsets vary in their interactions with Alzheimer's disease (AD) and Atherosclerosis. Neutrophils drive both AD and atherosclerosis through their aggregation and production of MMPs, ROS and NETs, which damage the blood brain barrier and heighten hypoxia-induced Aβ production, while the neutrophilia and increased activation of neutrophils seen in atherosclerosis may further feed into this damage. Monocytes phagocytize Aβ, but may also produce proinflammatory cytokines that drive harmful neuroinflammation, while the production of pro-inflammatory monocytes is increased in atherosclerosis, which accelerates atherogenesis. Both diseases drive differential production of classical and non-classical monocytes. TREM2+ macrophages may help to clear Aβ in the brain and mitigate inflammation in atherosclerotic lesions. Similarly, increased proportions of anti-inflammatory macrophage subsets appear to play a protective role in AD, while increased proportions of proinflammatory macrophages drive inflammasome activation and production of pro-inflammatory cytokines in atherosclerosis, causing plaque progression. Adaptive immune cells add further complexity to these diseases. The role of B cells in AD and atherosclerosis appears to be subset specific, with clearance of Aβ *via* antibodies tempered by the release of pro-inflammatory cytokines and differential effects by follicular B cells and innate response activator B cells on atherosclerosis. This is similar to the differential activity of T cells in these diseases. Controversy over the role of regulatory T cells in AD revolves around whether immune tolerance or immune activation is beneficial for AD. Th1 cells are prominent in progression of atherosclerosis and AD, producing high levels of IFNγ, which increases risk of plaque rupture in atherosclerosis and blood brain barrier breakdown in AD through increased activation of monocytes and macrophages and MMP release. Th17 cells are still largely understudied in AD, but their IL-17 production may contribute to AD possibly through increased migration of neutrophils and monocytes to the brain, although their role in atherosclerosis is still under investigation. Th2 cells do not appear to migrate to the brain, and studies differ on their role in AD, possibly due to the differential role of IL-4, IL-5, and IL-13 that they produce. This is mirrored in atherosclerosis, where IL-4 is atheropromoting, while IL-5 and IL-13 are atheroprotective, making for a complex relationship between Th2 cells and these diseases. CD8+ T cells are associated with AD progression through mechanisms that are still being explored, and may promote plaque instability in atherosclerosis.

Neutrophils, although crucial in acute immune assault, can prove to be highly damaging in the context of chronic inflammation presented in AD and atherosclerosis (Figure 1). In the case of AD, neutrophil accumulation and activation have negative consequences (42). Neutrophils from AD patients are highly activated and pro-apoptotic, produce intravascular neutrophil extracellular traps (NETs) and higher levels of ROS that correlate with amyloid burden (43). While precise mechanisms of neutrophil activation remain to be determined, it is likely that peripheral proinflammatory cytokines are instrumental in the activation of peripheral blood neutrophils. Neutrophil hyperactivation and impairments in homeostasis appeared to correlate with faster cognitive decline (43). In two transgenic models of AD (5xFAD and 3xTg-AD mice), neutrophils were found in Aβ-positive areas of the brain, where they released NETs and IL-17 (44). LFA-1-dependent infiltration of neutrophils were peaking at 4 months of age, which coincided with the onset of memory loss in cognitive tests of these animals (44). Neutrophil depletion in those mice improved performance in certain memory tests and reduced microgliosis highlighting a functional role of neutrophils in neuroinflammation. In further support of the negative effects of neutrophil aggregation, Cruz-Hernández et al. found that adhesion of neutrophils in the vasculature of the brain leads to reduced cerebral blood flow in APP/PS1 mice, which was subsequently resolved by treatment with anti-Ly6G antibody (Abs) (45). Another avenue through which neutrophils may further exacerbate AD is NETosis. Although NETosis can be activated through numerous mechanisms, of note, neutrophils interact with Aβ-activated platelets in the vasculature of the brain, an interaction that induces NETosis (46, 47). Increased NETosis in the small vessels of the brain promotes blood clotting through thrombin formation and further platelet aggregation, occluding the microvasculature and possibly furthering Aβ buildup through hypoxia-induced increases in β-secretase activity and reduced Aβ clearance (11, 48). NETosis can also damage the blood-brain barrier, further hampering the brain's clearance of Aβ (47). Proteases in these NETs such as MMP-9 also contribute to blood-brain barrier breakdown. In fact, a recent study by Ringland et al. found that inhibition and/or knockout of MMP-9 resulted in reduced anxiety and improved social recognition memory in an AD mouse model, despite no reduction in Aβ levels (49). Thus, burgeoning data demonstrate that neutrophils are a significant component of the immune response that is associated with neuroinflammation and have an instrumental role in AD pathophysiology.

The pathological role of neutrophils in atherosclerosis has been firmly established and data indicate that chronic inflammatory environment in atherosclerosis shapes number and phenotype of circulating neutrophils, which has been reviewed elsewhere (50). Pro-atherogenic environment impairs cholesterol efflux, induces inflammasome activation, alters the balance of myeloid and lymphoid hematopoietic stem and progenitor cells in the bone marrow, and results in enhanced myelopoiesis and elevated number of neutrophils and monocytes in the blood (51, 52). It is likely that the increased neutrophil production observed in atherosclerosis may serve to facilitate further neutrophil aggregation in the brain, and future studies using adoptive transfer recruitment experiments could answer this important question.

**Figure 2:**
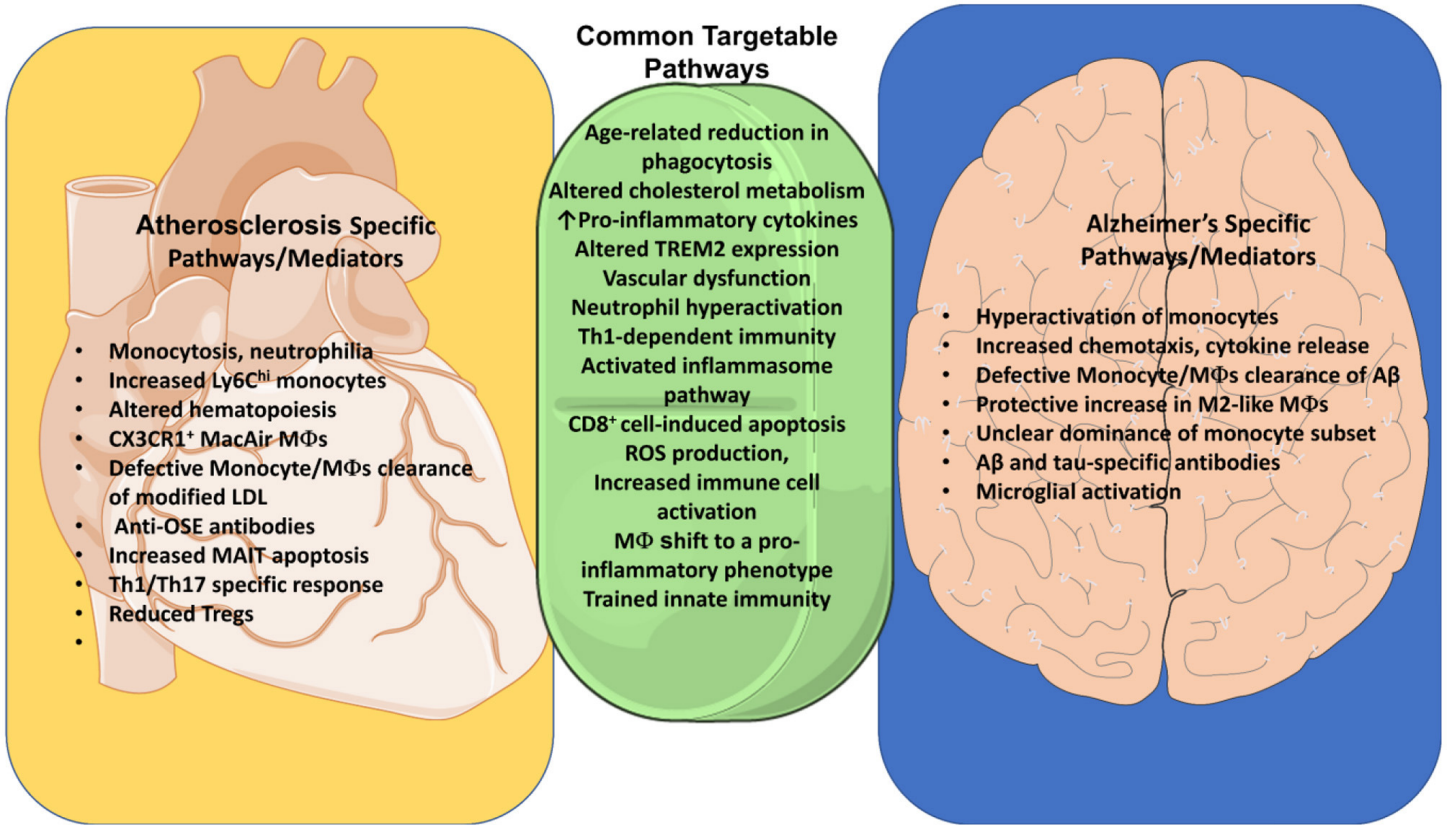
Common pathways of Immune dysregulation between Alzheimer's and Atherosclerosis. Identification of common pathways between AD and atherosclerosis identifies targets for treatment of both diseases. Both diseases are influenced by an age-related reduction in phagocytosis, as well as cholesterol metabolism, vascular dysfunction, activation of the inflammasome pathway and ROS production. Neutrophil hyperactivation and Th1-dependent immunity are both detrimental to these diseases. TREM2 assists myeloid cells in clearance of Aβ and cholesterol in their respective diseases. Both diseases also share an increase in IL-4, as well as apoptosis of endothelial cells by CD8+ T cells. We additionally explore differences between these diseases such as the production of OSE-specific antibodies, increased monocytosis, increased Ly6C^hi^ monocytes, increased apoptosis of MAIT cells, and differentiation of Mac^AIR^ macrophages in atherosclerosis. Alzheimer's, in contrast, features a clearance of Aβ by monocytes and macrophages, activation of microglia, as well as Aβ- and tau-specific Abs. Some of these differences may with time be challenged as we uncover more commonalities and ties between these diseases.

Not only number, but the activated phenotype of neutrophils in atherosclerosis might result in collateral damage from their effector functions within various tissues including the brain (Figure 1). Several studies demonstrated that increased ROS production by activated neutrophils is implicated in endothelial dysfunction and plaque instability (50). As neutrophils are found in the vasculature of the brain, it is likely that these activated cells may induce vascular cell apoptosis and dysfunction. Neutrophils are also known to contain a large pool of granules such as azurocidin, proteinase 3, α-defensin, myeloperoxidase, LL37 (CRAMP in mouse) and Cathepsin G, which serve as activators of endothelium and some as chemoattractants for monocytes. These active compounds are also known to increase the expression of adhesion molecules and chemokine receptors in endothelial cells and regulate endothelial cell permeability in mice. Taking into an account a pivotal role of neutrophils in regulation of monocyte recruitment, it would be imperative to test the implication of neutrophils in modulation of leukocyte, and particularly, monocyte recruitment into inflammatory brain tissues at different time points of AD development. Neutrophils are found within atherosclerotic lesions and are believed to be responsible for many proinflammatory processes leading to a formation of unstable plaques (2, 50). Once homed to plaques, neutrophils degranulate and release NETs, MPO, proteases such as MMP-9, which contribute to additional ROS production, thinning of the fibrous cap, and ultimately plaque instability and rupture (50). Circulating NET markers have also been identified as risk factors for CVD severity (2). Recently, Silvestre-Roig et al. demonstrated that NET-released histone H4 can bind to and lyse vascular smooth muscle cells (VSMCs), leading to their apoptosis and plaque destabilization, highlighting a novel role of neutrophils in atherosclerotic plaque burden and stability that may additionally be explored in the blood-brain barrier breakdown seen in AD (53). NETs can prime macrophages to produce IL-1β *via* an inflammasome–dependent mechanism (54) in atherosclerosis and activated neutrophils may pulse microglia to over-reactive activated phenotype as well. Based on the critical functions of neutrophils in AD and their increasing numbers and pathological functions in atherosclerosis, it is clear that neutrophils and particularly neutrophil-derived NETs might be one of the important key players in AD and associated atherosclerosis (Figure 2). To date, several questions require additional studies in order to better understand triggers and mechanisms by which neutrophils can be activated in AD and associated CVD. Neutrophil-derived NETs can interact with monocytes/macrophages, dendritic cells, and platelets, regulate their functions, and accelerate damage of surrounding tissues. What is not well yet known how NETs can regulate neuroinflammation and particularly the impact of NETs on health and functions of microglia as well as vascular cells including pericytes, VSMCs, and endothelial cells and additional studies are warrant in this direction. Taken together, current findings indicate that neutrophils fuel central processes supporting both atherogenesis and AD; thus, targeting pathways of pathological neutrophil activation such as NET formation, may be a promising therapeutic approach that would have a double effect.

## Monocytes and Macrophages in AD and Atherosclerosis

The role of monocytes, macrophages and microglia in AD development is a topic of extensive studies (6, 10) and epidemiological and experimental data reveal differential roles of microglia, monocytes and cerebral macrophages in Aβ pathophysiology (55). The significance of microglia and macrophages in systemic inflammation and neurodegenerative diseases have been discussed comprehensively by others (55). In this review, we focus on main points of monocyte/macrophage phenotype in AD that has some commonality with described functions of monocytes/macrophages in atherosclerosis (Figure 2).

There are distinct quantitative and qualitative changes in monocytes as AD progresses with some controversial results in the field (6, 10). Evidence suggests that AD patients demonstrated a decrease in the non-classical CD14^+^CD16^++^ monocytes in comparison with patients with mild cognitive impairment or healthy controls (56). In contrast, Munawara et al. reported an increases in the percentage of non-classical and intermediate monocytes and the decreased percentage of classical monocytes in the blood of AD patients vs. healthy controls (57). Importantly, several studies also reported an overall increase in circulating monocytes in AD that exhibit hyperactivated phenotype characterized by increased chemotaxis, ROS production, and cytokine release in response to TLR2 and TLR4 stimulation (57). Importantly, these features of activated phenotype are a common denominator for all reported studies on monocyte phenotype in AD pathology.

Monocytes and monocyte-derived macrophages appear to play a complex role in AD, as these cells help to phagocytize and thereby clear the Aβ in AD patients while simultaneously arousing increased inflammation through release of potentially damaging proinflammatory cytokines such as IL-1α/β, IL-6, TNFα, IL-8 and TGFβ (5). Thus, emigrated monocytes perform dual role within the brain exacerbating or alleviating disease progression. The importance of recruited monocytes in the regulation of neuroinflammation has been elegantly demonstrated in experiments with CCR2-deficient mice (58). Naert et al. showed that an impairment of *Ccr2*^−/−^ monocyte trafficking to the brain results in increased levels of intracellular Aβ as well as worsening of cognitive impairment in APP/PS1 mice (58), suggesting the overall protective role of CCR2+ monocytes in AD. Additionally, Naert et al. found deficient production of classical CX3CR1^low^Ly6-C^high^Gr1+CCR2+ monocytes in the bone marrow of 6-month-old APP/PS1 mice, whose rescue through treatment with M-CSF prevented cognitive decline (59).

As BM-derived macrophages are known to be more efficient than resident microglia in clearing Aβ deposits in AD models (60), it appears that this is a key decisive function of monocyte in the regulation of neuroinflammation. Here, it is important to note that monocyte phagocytic capability becomes compromised with age. Age correlates with reduced phagocytosis of Aβ in both classical CD14^+^CD16^−^ and non-classical CD14^dim^CD16^+^subsets (61). This reduction was concomitant to diminished chemotaxis in response to CCL2 in patients with AD and reduced phagocytic capacity of monocytes in patients with mild cognitive impairment compared to healthy controls (57). Subsequent differentiation into monocyte-derived macrophages using the patients' sera resulted in increased proportions of M2-like vs. M1-like macrophages in AD patients (57). These M2 macrophages may be playing a protective role, as transplantation of M2 macrophages into Aβ-treated rats improved performance in cognitive testing, and in APP/PS1 mice reduced Aβ plaque size (62, 63). Collectively, these data point out the importance of phagocytosis and proper clearance of Aβ and damaged cells at different stages of AD development as a combination of defective phagocytosis and inflammatory insults deadly affects microglia health and functions. Indeed, several studies now demonstrate that the inhibition of key inflammatory mediators improves AD pathology and emphasize a critical role of microglia in this process (10, 64). In these regards, studies investigating a role of microglial surface receptor TREM2 helped to dissect an impact of functional microglia in AD. These reports showed that overexpression of TREM2 improves the cognitive decline in mouse models of AD (65) and TREM2 deletion aggravates pathology *via* regulation of macrophage and neuronal phenotypes and the Aβ uptake (66). While significant efforts have been directed to examination of microglia and blood-derived macrophage functions in AD, few studies have been devoted to a role of the specific population of macrophages surrounding brain vessels - perivascular macrophages in AD. Depletion of perivascular macrophages significantly increased the number of Aβ+ cortical blood vessels. Conversely, stimulation of perivascular macrophage turnover reduced cerebral amyloid load (67). In contrast, Park et al. reported that a selective depletion of perivascular macrophages abrogates cerebrovascular dysfunction and CD36- and Nox2 expressing by macrophages are responsible for pathological effects of perivascular macrophages (68).

The role of monocytes and macrophages in atherosclerosis have been described in many excellent reviews (4, 69, 70). In this review, we highlight features of monocyte/macrophage responses in atherosclerosis that might have a potential implication in modulation of AD pathophysiology (Figure 1). One of the important factors that is critical for the AD acceleration in atherosclerosis is the impact of pro-atherogenic conditions including hyperlipidemia on circulating monocyte numbers. Hematopoietic stem and progenitor cells (HSPCs) demonstrate reduced regenerative capacities and a shift toward myeloid cell differentiation in atherosclerosis (71) resulting in monocytosis with a dominant increase in pro-inflammatory Ly6C^hi^ monocytes (72–74) that is linked to accelerated atherosclerotic lesion progression in mice as well as increased risk of CVD in humans (72–75).

Macrophages are professional phagocytic cells that uptake lipoproteins and form lipid-enriched foam cells (4). While it was initially thought that lipid uptake results in pro-inflammatory phenotype of macrophages, recent data point out that foam cell formation does not result in a pro-inflammatory phenotype of macrophages (76, 77), but pro-inflammatory conditions reverse the phenotype of these macrophages. The impact of cholesterol on monocyte/macrophage biology in AD and associated atherosclerosis is not well-understood (78). Several studies revealed harmful effect of dyslipidemia on AD. A number of genes involved in cholesterol metabolism or transport are AD susceptibility genes, including apolipoprotein E (*APOE*), apolipoprotein J (*APOJ, CLU*), ATP-binding cassette subfamily A member 7(*ABCA7*), and sortilin-related receptor (*SORL1*) has been identified as a pivotal components of cholesterol metabolism in the brain (78). Epidemiological studies detected an association between dyslipidemia and AD and clinical trials demonstrated some controversial effects of statin therapy AD (79, 80). As cholesterol loading alters membrane integrity, one of the interesting questions that has to be addressed in the future experiments is how increased cholesterol levels affect ability of monocyte-derived macrophages and microglia response to inflammatory insults and effectively phagocyte accumulating Aβ.

Macrophage polarization is also greatly affected by atherosclerosis and a broad spectrum of macrophages with either proinflammatory or anti-inflammatory features is found within the aortic wall. To date, there are at least five subsets of macrophages detected with the aortic wall (81). Classical inflammatory macrophages display an activated inflammasome pathway and express a myriad of pro-inflammatory cytokines and chemokines. Atherosclerosis-induced subset of macrophages that express various interferon-inducible genes, including *Ifit3, Irf7 and Isg15* is also identified in the lesions (81). A subset of pro-inflammatory macrophages also originate from CX3CR1+ precursor cells in the aorta. Characterized by a specific location with the intima, aortic intima-resident macrophage (Mac^AIR^) subset have a pro-atherogenic role at the early lesion development stage. The most interesting population in connection with AD is lipid-loaded macrophages expressing TREM2 that have been found within plaques at different stages of atherogenesis (60). TREM2+ macrophages have differentiated either from circulating monocytes or from embryonic precursors, express cholesterol metabolism-related such as *Cd9, Ctsd, Fabp4 and Abcg1*, which have roles in lipid metabolism, cholesterol efflux and oxidative phosphorylation. TREM2+ macrophages represent a major population of lipid-filled macrophage-derived foam cells, unexpectedly express low levels of inflammatory genes, and are postulated to have a role in mitigating vascular inflammation (76, 77).

As they are brain-resident cells, microglia in particular are not necessarily of interest in atherosclerosis. However, cells in atherosclerosis do have parallels to disease-associated microglia's (DAMs), and atherosclerosis in general can affect the behavior of microglia (55, 60). The lipid droplets in DAMs are reminiscent of lipid accumulation in foam cells during atherosclerosis and transcriptomic analysis has found enrichment of foam-cell enriched genes in DAMs (82). Aβ-laden DAMs downregulate expression of *P2ry12, Cx3xr1*, and *Tmem119* while upregulating genes such as *Apoe* and *Trem2* (83). These DAMs initially play a protective role, phagocytizing Aβ through upregulated TREM2 without significant inflammation, but in later stages of AD these DAMs become more inflammatory (83, 84). Yeh et al. also found that TREM2 serves as scavenger-like receptor that binds to lipoproteins associated with Aβ, which allows the microglia to uptake Aβ more efficiently (85). It might be possible that TREM2 serves as a key role in the anti-inflammatory properties of TREM2+ microglia and TREM2+ macrophages in the aortas. Since part of TREM2+ aortic macrophages are differentiating from monocytes, it might be beneficial to design studies that would identify pathways leading to a generation of monocyte-derived TREM2+ macrophages and a way how direct this population to migrate to the brain tissues for effective efferocytosis and suppression of neuroinflammation.

It is interesting to note that similar to a shift from initially protective macrophages to pro-inflammatory macrophages in AD, shift from protective/clearing functions of macrophages is also observed in atherosclerosis providing an additional line of similarities and potential therapeutically targeted points for these two diseases (Figure 2). In both pathologies, macrophages (and microglia in AD) play a multidimensional role *via* clearance of dying cells, processing its metabolites, taken up lipids, secretion of cytokines/chemokines and MMPs and therefore regulation of additional efflux of immune cells and regulation of vascular cell functions. On the other hand, macrophages can also promote resolution of inflammation. Thus, a therapeutic goal may be to find a network that would support “Golden Mean” of macrophages and microglia between their inflammatory functions, proteolytic activities and housekeeping functions of proper cholesterol transport, cellular flux, and effective efferocytosis in both pathologies.

Emerging evidence indicates that innate leukocytes can acquire an innate cell memory that is characterized by enhanced responsiveness to activation triggers. Innate trained memory is formed as a consequence of reprogramming based on gene transcription changes, epigenetic processes, and altered cellular metabolism (86). While more evidence is needed, initial studies demonstrate an existence of innate trained immunity in both atherosclerosis and AD (87, 88). It is likely that common triggers of innate memory such as infections in conjunction with the disease-specific stimuli including oxLDL, LDL and high-mobility group box 1 (HMGB1), can induce trained immunity in atherosclerosis (88). While it is unclear whether endogenous stimuli can induce trained innate immunity in AD, several infections such as Herpes simplex virus type 1, Cyto-megalovirus, Chlamydophila pneumoniae, spirochetes, Helicobacter pylori, and periodontal pathogens are closely associated with AD (89), suggesting these pathogens can serve as triggers of innate memory in AD patients. What are the targets for trained immunity? It has been reported that bone marrow progenitors, mature innate immune cells, microglia as well as NK cells, endothelial and VSCMs can develop memory characteristics. A common signature of innate trained immunity defined as an elevated long-lasting altered inflammatory response is detected in myeloid cells from patients with AD and/or atherosclerosis (87, 88). Indeed, elevated levels of circulating pro-inflammatory cytokines and amplified monocyte/macrophage cell responses have been reported in AD patients (27, 90). Not only AD circulating monocytes/neutrophils but also AD microglia demonstrate an activated phenotype that is consistent with a phenotype of innate memory response with a signature of alterations in cell transcriptome, epigenetic landscape and metabolic phenotype [reviewed in (87)]. Interestingly, in the aging brain and more evidently in AD patients, microglia appear primed: microglia are activated, produce increased amounts of pro-inflammatory mediators and are more susceptible to central damage after peripheral insults (93, 94). Interestingly, circulating pro-inflammatory cytokine levels are also dependent on the stages of disease progression with an overall reduction at the severe AD stages (91), suggesting a dynamic recalibration of the trained innate response likely due to the induction of tolerance.

In line with the trained immunity phenotype in AD, several papers highlighted a signature of trained immunity in atherosclerosis, particularly to various microbial and atherosclerosis-related stimuli in the *in vitro* system with a macrophage response characterized by elevated levels of TNFα, IL-6, MCP-1, MMP-2 and MMP-9, and increased foam cell formation (92). *In vivo* mouse studies showed that 4 weeks of LPS-induced TLR4-engagement initiated a reprogramming of monocytes to an inflammatory phenotype and aggravated atherogenesis (93). A similar signature of trained immune response with an increased *ex vivo* cytokine production and an elevated endothelial cell adhesion and migration was observed in patients with atherosclerosis (94). Not only elevated production of pro-inflammatory cytokines, but also evidence of metabolic reprogramming and epigenetic modifications at the level of histone methylation has been detected in blood monocytes of patients with established atherosclerosis (95). Thus, trained innate immunity is active in both conditions of AD and atherosclerosis and may contribute to the regulation of dysbalanced innate response that has a key role in driving both pathologies. While the majority of studies in both AD and atherosclerosis are focused on the characterization of the innate cell phenotype, very little is known about effects of innate memory responses on other myeloid cell functions such as recruitment, efferocytosis, apoptosis, and DC and macrophage antigen presentation and further studies are necessary in this promising area. It would be also critical to identify key characteristics of a monocyte population, conditions, and mechanisms by which monocytes might be recruited to inflammatory brains at different time points of the AD progression. As AD and atherosclerosis are diseases of aging, it would be further important to develop research programs focused on mechanisms that connect trained immunity and “inflammageing”, particularly in atherosclerosis-associated AD. These studies would help to better understand effects of trained innate memory immune responses in AD and atherosclerosis.

## B Cells in Alzheimer's Disease and Atherosclerosis

While the prominent role of the innate immune system is extensively studied in AD, the role of adaptive immune response, and particularly B cells, in this disease is not well-understood, but an increasing body of evidence suggests an implication of B cells in AD (96). Indeed, one of the key discoveries in B cell–related pathophysiology of AD relies on finding that Aβ and tau are immunogenic proteins that induce an Ab response in AD (97). Importantly, these anti-Aβ (98) and anti-tau (99) Abs can bind its antigens in brain tissues of AD patients. In line with the observation of specific Abs against Aβ and tau, the analysis of the BCR repertoire revealed an amplification of high-frequency clonotypes in AD vs. healthy subjects suggesting that BCR-antigen interactions lead to a clone expansion (100). It remains to be determined specific functions of anti-Aβ and anti-tau Abs *in vivo* as followed up studies provided controversial conclusions for a role of Ab-specific Abs in AD pathology (Figure 1).

Studies in mice suggest that Igs play a dual role in AD since they activate microglia phagocytosis reducing Aβ deposits, but can also induce over-activation of microglia and neuronal neurotoxicity (101). Specifically, in 5 × FAD mice, Rag deficiency exacerbates AD due to loss of non-specific Igs that activate microglial phagocytosis and consequent clearance of Aβ plaques (101). Several studies attempted to use anti-Aβ Abs to reduce progression of AD in humans (102). Intravenous immunoglobulin (IVIG), a mixture of healthy donor-derived polyclonal Abs that contains most of the IgG found in the human immune repertoire, have been used in AD patients and showed some beneficial effects. Not only passive administration of Aβ-specific Ab, but also active immunization with Aβ reduced Aβ deposits in AD patients (103, 104) and mice, ameliorating the severity of disease (105, 106). The immunization with Aβ_1−42_ reduces Aβ plaque burden and preserves cognitive function in APP transgenic mice. Subsequently, this approach has been used in a clinical trial of AN1792(QS-21) in patients with mild to moderate AD. The study was interrupted because of development of meningoencephalitis in 6% of immunized patients, but initial data on neuropsychological test battery revealed differences favoring Ab responders (103) suggesting a protective role of Igs against Aβ in AD progression.

B cells not only produce Igs but also serve as antigen-presenting cells and cytokine-producing cells (16). Recent study by Kim et al. focused on the overall B cell functions in AD and demonstrated that the loss of mature B cells alone is sufficient to markedly retard the AD progression, improve behavioral and memory capacities and restore TGFβ+ microglia in the transgenic AD mouse models (107). Another function of B cells is to regulate inflammation *via* production of cytokines and chemokines. In line with a pathological function of B cells, B cells from AD mice acquire an inflammatory phenotype as shown by the upregulation of pro-inflammatory cytokines, including IL-6, TNFα, and IFNγ (42). Thus, B cells might play different roles in AD and associated neuroinflammation *via* secretion of immunomodulatory molecules, such as cytokines or Abs in periphery or directly affect glial reactivity and induce neuronal damage by secretion of inflammatory proteins at the site of damage *via* migration through dysfunctional blood-brain barrier.

B cells are active players not only in AD, but also have a prominent subset-specific role in atherosclerosis (16, 108). B1 cells synthesized natural Abs against pathogen-associated molecular patterns and neo-antigens. MZ B cells protect against blood-borne pathogens. FO B cells are activated by antigen stimulation, undergo germinal center reactions, and differentiate into memory B cells or plasma cells. Overall, FO and IRA B cells support atherosclerosis development, while B1 and MZ B cells protect against atherogenesis (45, 109). The role of B cell subsets in AD is not yet investigated, but some evidence suggests that it could be B cell subset specific. For example, several studies showed that neutralizing IgGs against Aβ are protective against AD, suggesting a potential role of FO B cells in AD (110, 111). Interestingly, a wide range of auto-Abs against Aβ, tau, neurotransmitters and related receptors, lipids, as well as microglial markers, presumably produced by B1 cells, are found in AD patients as well as healthy subjects (112). It is possible that activated upon atherogenic conditions in atherosclerosis, FO B cells can provide a further set of Ab-producing cells, while activated B1 cells might release Abs with a specific to neo-antigens including the Aβ protein. Further studies are necessary to determine which Ab isotypes are dominant in AD, characterize antigen-specificity of the response, and finally identify a role for germinal centers in driving AD pathologies. Taking into account some similarities in the nature of the diseases that are characterized by the chronic inflammatory response associated with aging and responses to self-antigens, it might be possible that FO, MZ, and B1 cells play a diverse role in AD. As atherosclerosis shapes number and functions of B cell subsets, it is likely that B cell subsets might display a unique phenotype and functions in atherosclerosis-associated AD and further studies will be necessary in this direction.

## T Cells in Alzheimer's Disease and Atherosclerosis

Like B cells, T cells appear to have a complicated relationship with AD that is heavily T cell subset dependent (42). Although the role of T cells in AD is still being explored, there has been extensive effort into identifying changes in T cell subsets with varying degrees of agreement among studies. CD4^+^ T effector cells vary drastically in their role in AD. Adoptive transfer of Aβ-reactive Th1 or Th17 cells into the APP/PS1 mice led to an increase in Aβ in the brain through downregulation of Tregs (113). Additionally, IFNγ produced by adoptively transferred Aβ-specific Th1 promoted increased Aβ deposition in an APP/PS1 model, alongside cognitive deficits, indicating a detrimental role for Aβ-specific Th1 cells in AD (114). In line with these data, T cell infiltration into the brain is increased in AD compared to steady-state conditions, and studies in humans have found increased levels of Aβ-reactive T cells in AD patients and older adults compared to younger, healthy controls (115).

Circulating Th17 cells are increased in mild cognitive impairment (MCI) compared to cognitively normal controls (116). Neutralization of IL-17 prevents short-term cognitive deficits in an AD mouse model and attenuates deficits in long-term potentiation, although the primary source of IL-17 appeared to be from γδ T cells (117). One of the potential mechanisms of pathological action of infiltrating Th17 cells is upregulation of neuron-associated Fas and Th17-associated FasL, possibly contributing to increased neuronal apoptosis (118). Th2 cells appear to play a vastly different role in the AD-associated adaptive immune response. Adoptive transfer of Aβ-stimulated Th2 cells reversed cognitive impairment, reduced plaque associated microglia, decreased cerebrovascular Aβ deposition, and returned plasma cytokine level to normal levels in a mouse model (119). Specifically, lowered circulating GM-CSF correlated greatly to behavioral improvement, implying Th2 cells may soothe myeloid-induced neuroinflammation (119). In contrast, Baruch et al. found elevation in IL-4 expression in the choroid plexus of older mice coincided with production of CCL11, a chemokine associated with cognitive decline suggesting a pathological role of Th2 in neuroinflammation (120).

CD8+ cells have long been known to infiltrate into the brain during AD (121). Recent studies showed significantly more activated CD8^+^ T cells, particularly CD8^+^CD27^−^ Teffector memory CD45RA^+^, in the blood and cerebrospinal fluid of patients with mild AD compared to healthy controls with a positive association with neuropsychological deficits, suggesting a detrimental role for CD8^+^ cells in AD (122, 123). In a mouse model of AD, doublecortin^+^ CD8+ cells clustered at Aβ plaques, and ablation of CD8^+^ T cells from the blood and brain resulted in changes in neuronal- and synapse-related gene expression (124). This depletion did not improve either cognition or Aβ pathology, although these mice were treated at 12 months of age, and may have already undergone extensive irreparable neurodegeneration. It would be important to understand mechanisms of doublecortin^+^ CD8+ cells recruitment to the aged brain as these cells can be a key participant in the regulation of synaptic plasticity.

Recent studies have unveiled a significant controversy in the trend for numbers of peripheral Tregs with decrease, increase or unchanged levels of Tregs in AD patients compared to patients with mild cognitive impairment or healthy controls (125, 126). This discrepancy could be due to differences in patient categorization of patient populations or gating strategies in flow cytometry. Mouse studies into the role of Tregs have caused even further confusion, as controversies arise over whether the immune-suppressive nature of Tregs propagates or protects against neurodegeneration. The adoptive transfer of Tregs into 3xTG mice reduced Aβ burden in the brain and improved spatial learning memory while reducing production of IL-2, IL-6, and IFNγ and increasing IL-10 production (127). In contrast, the depletion of Foxp3^+^ cells reduced Aβ plaque burden in 5xFAD mice (128). In line with pathological role of Tregs, induction of IL-10 in the brain using AAV vectors injected into the cerebral ventricles increased Aβ deposition and worsened fear-conditioned memory (129). Altogether, growing data indicate that T cell subsets are involved in AD-associated pathology and surprisingly demonstrate an altered distributions and activation statuses, thus playing a T cell subset specific role in this pathology (42). The ways in which peripheral T cells in AD showed a high sensitivity to peripheral inflammatory mediators suggest that these cells would be highly susceptible to a Th1/Th17-associated atherogenic response upon atherogenesis.

The overall T cell implication in atherosclerosis and their subset-specific roles have been discussed in several recent reviews (4, 130). Here, we focus on potentially important features of T-cell response in atherosclerosis that could affect the response in AD (Figure 1). Th1 cells are the predominant pathogenic T cell in atherosclerosis, contributing to high levels of IFNγ, which activate macrophages and monocytes and upregulate expression of MMPs, thinning the fibrous cap and increasing risk a plaque rupture. Similar to AD, Th1 cells are associated with worsening of atherosclerosis, as exogenous IFNγ enhances atherosclerosis in *Apoe*^−/−^ mice (4, 131, 132). An important study using Tbet, a transcription factor responsible for a Th1 differentiation, showed that Tbet deficiency caused a Th2 switch, changed in T-dependent isotypes of oxidized LDL-specific Abs and reduced atherosclerosis (133). How elevated levels of Th1-derived cytokines modulates AD neuroinflammation remains to be determined; however, initial studies, particularly revolving around IFNγ, demonstrated somewhat diametrically opposed functions in the setting of AD-related neurodegeneration (134).

The role of Th17 cells in atherosclerosis is complex, and seems to be dependent on the model of atherosclerosis and timing of measurements of plaque development (135). One of highlighted features of IL-17+ T cells is a capacity to enhance endothelial expression of P-selectin, E-selectin, CXCL1, CXCL2, and CXCL5, potentially increasing migration of neutrophils and monocytes to the tissue specific site (136). This implies that Th1 and Th17 (or other IL-17 producing T cells) could act synergistically to further support recruitment of monocyte and neutrophils in the brain. Tregs are atheroprotective, with their expansion by various means detailed in previous reviews (130) resulting in reduced plaque formation due to the release of anti-inflammatory cytokines IL-10 and TGFβ. In the context of AD and atherosclerosis, modulation of Tregs or their downstream mechanisms could abate the damaging aspects of inflammation seen in both diseases, although more information is needed in regards to the role of Tregs as a whole in AD. Th2 cells are rarely found in atherosclerotic lesions, and are controversial in their role in atherosclerosis, much like in AD (1, 3). Deficiency in IL-4, a Th2 cytokine, in *Apoe*^−/−^ mice reduced plaque area, implicating it in atherosclerotic progression, while the Th2 cytokines IL-5 and IL-13 appear to be largely atheroprotective (3). CD8^+^ cells are present within atherosclerotic lesions, with many of these identified as CD45RO- and very late activation antigen-1-expressing memory T cells (137). CD8^+^ T cells persist in the fibrous caps of plaques, and may contribute to plaque instability and rupture by inducing apoptosis of endothelial cells. In addition to their influence on monocytes and neutrophils, T cells may also be altered in terms of innate immunity. Mucosal-associated invariant T cells (MAITs) are thought to be involved in as well as influenced by immune-mediated chronic diseases, including type 2 diabetes mellitus and coronary artery disease (138, 139). Touch et al. found that individuals with coronary artery disease had decreased circulatory levels of MAITs with increased proapoptotic signaling compared to healthy controls, which may be caused in part by the chronic inflammation associated with the disease (139). Although the role of MAITs in AD has remained relatively unexplored, MAIT cells are known to be present in the brain, where they may interact with microglia and astrocytes *via* the major histocompatibility complex class Ib molecule called MR1 (140). Circulating levels of MAIT cells have been found to decline in later life (141), an observation whose explanation may further elucidate what role these cells play in atherosclerosis and AD. T cell subsets play an integral role in both AD and atherosclerosis, although whether this role is protective or harmful remains to be fully elucidated in many subsets. Given the fact that T cells are rarely found within the healthy CNS parenchyma, it would be important to investigate when, how and which subpopulation of Th cells can migrate to the brain tissues and regulate the local microenvironment. It is also important to determine whether only CNS antigens- specific Th cells or a broader range of CD4+ effector memory cells might be recruited and modulate the local immune response in the brain under conditions of atherosclerosis. Increasing evidence indicates that homeostasis of peripheral T cell immunity is dysregulated in AD and atherosclerosis and these changes in T cell subset phenotypes and activation status affect the brain and vessel biology and have complex implications in both disease development and persistence. Gaining an understanding as to how proatherogenic Th cells modulate the AD-associated response could elucidate new therapeutic strategies designed to harness the activity of pathological Th subsets in both AD and atherosclerosis.

## Hypercholesterolemia in the Crossroad of Atherosclerosis and AD

Hypercholesterolemia has long been agreed upon as a risk for atherosclerosis, but hypercholesterolemia could also play a pivotal role in neurodegeneration in AD (Figure 2). Twenty-five percentage of the body's cholesterol is located in the brain, where it is synthesized *de novo* due to its inability to cross the brain blood barrier (11). Despite this fact, a meta-analysis of vascular risk factors in AD found that systemic hypercholesterolemia increases the risk of AD (142), and higher levels of LDL have been associated with higher Aβ burden in autopsies of AD patients independent of APOE status, indicating a link between peripheral hypercholesterolemia and AD. This link may in part be driven by the effects of hypercholesterolemia on peripheral immune cells. Hypercholesterolemia induces monocytosis and neutrophilia by increasing expression of CCR2 and CXCR2, promoting atherosclerosis but also potentially contributing to brain blood barrier breakdown and immune infiltration (143). Brain blood barrier breakdown increases the egress of 24S-hydroxy-cholesterol, a metabolite of cholesterol that modulates cholesterol synthesis, which in-turn increases the production of cholesterol in the brain (11). Increased neuronal cholesterol increases amyloidogenic processing of APP through assembly of APP, β-secretases, and γ-secretases in lipid rafts (125). Similarly, hypercholesterolemia increases TCR signaling through increased assembly of lipid rafts, which help in the dimerization of TCR (144). This leads to hyperproliferation in CD4+ T cells, which could serve to exacerbate AD (10). Evidence suggests that lymphatic functions are disregulated under hypercholesterolemic conditions and dysfunctional lymphatics are likely involved in atherogenesis (145). Importantly, meningeal lymphatics have been recently discovered across the species and its role has been implicated in development of several neurodegenerative pathologies including AD (145). Therefore, there is a need in new studies focused on a potential implication of hyperlipidemia in the regulation of lymphatics in AD that might open new strategies for combatting both AD and atherosclerosis. One of the strongest links that ties AD and atherosclerosis is the fact that the APOE4 isotype is a risk factor for both hypercholesterolemia and AD (11). APOE transports cholesterol-carrying lipoproteins from astrocytes to neurons in the brain (11), but it is still unclear how differences in APOE isoforms in the brain mediate increased prevalence of AD. In the periphery, the APOE4 isoform may contribute to AD through increased prevalence of hypercholesterolemia and plasma LDL levels, which may then indirectly modulate AD through changes in immune response. Therefore, although many mechanisms modulate the interconnections interwoven between atherosclerosis and AD, hypercholesterolemia-induced immune perturbation stands as one of a driving force that needs further exploration.

## Discussion

Although the brain has previously been considered an immune privileged organ, it is apparent that the peripheral immune system plays a large part in the brain's diseased state. Aging leads to chronic, low-grade inflammation that underlies the pathogenesis of many aging-related diseases, like AD and atherosclerosis, in a process known as inflammageing. Current work in AD focuses mainly on changes in local immune response, while atherosclerosis is known for its unique strong systemic immune response. Based on epidemiological data, AD and atherosclerosis as well as other chronic inflammatory diseases are interlinked, indicating a need for animal models that may elucidate the effects of atherosclerosis-derived systemic immune changes on AD-linked neuroinflammation. More exploration into how chronic peripheral inflammation, such as in atherosclerosis, may alter, exacerbate, or even cause AD may help to further reveal the roles of immune cell subsets in both diseases. Our current understanding of the role of neuroinflammation in AD is very reminiscent of atherosclerosis. Drawing parallels to compare DAMs and Aβ to foam cells and oxLDL has revealed that some of the same underlying mechanisms may be at play, and more similarities may be discovered in future studies. Clarification of the effects of peripheral inflammation on long-term plasticity in the function of the resident brain immune cells, like microglia, is needed to understand the changes in these cells. Additionally, as microglia are long-lived cells, there exists potential for innate trained immunity in the brain that has been explored in recent years (86, 146). Further study into how epigentic changes in atherosclerosis-influenced trained immunity of peripheral immune cells may contribute to AD will additionally shed light into how these two diseases may interact. Although not the central focus of this particular review, both AD and Atherosclerosis share common attributes of endothelial dysfunction and vascular aging, as well as shared risk factors that may contribute to the altered immune landscape. Aging, obesity, type-2 diabetes, and smoking are just a few inflammation-inducing risk factors that AD and atherosclerosis share. Aging of vasculature itself can lead to increased vascular stiffness, increased immune infiltration due to increased expression of leukocyte adhesion molecules on endothelial cells, and increased release of proinflammatory cytokines by immune, endothelial, and VSCMs [further reviewed elsewhere (147, 148)]. Endothelial cells become more senscent-like with age, with increased activation of NF-κB and apoptosis that may play a role in the recruitment of immune cells to the vasculature (149). Therefore, aging of the vaculature and endothelial dysfunction serve as an additional link between these two diseases.

While we have mainly focused in this review on the similarities and intertwined nature of the immune response in AD and atherosclerosis, another important aspect of the discussion should be an implication of gut microbiota and associated immunity in development of both pathologies.

Dysbiosis of gut microbiota has been reported to induce and support the progression of AD reflected by formation of Aβ plaques and neurofibrillary tangles. From the other hand, studies have also demonstrated that alterations in gut microbiota induced by a number of factors can lead to leaky gut, causing an increased inflammation and accelerated atherosclerosis. Thus, gut microbiota and gut-associated immune responses might have a synergistic effect on development of both AD and atherosclerosis. For a more detailed overview of how gut microbiota influences atherosclerosis and AD, we refer to related reviews (150, 151).

Current studies of AD in humans face challenges in unraveling the temporal and spatial aspects of immune infiltration in AD progression. Epidemiological data focuses on either collection of peripheral samples (such as blood and cerebrospinal fluid) in living humans or evaluation of the brain *via* imaging and/or post-mortem evaluation. New tools may be needed to identify where and when infiltration of the brain by immune cells occurs, perhaps through further development of imaging technology like two-photon microscopy. Although this review has encapsulated many aspects of the immune system in atherosclerosis and AD, it is limited in its discussion of other factors, such as an impact of oxidative stress, hypertension, hyperlipidemia and other metabolic aspects that have led some to refer to AD as type 3 diabetes (152). A detailed knowledge of immune pathways in atherosclerosis and AD and their interconnected molecular networks may also lead to a better understanding and treatment of both pathologies simultaneously, reducing a harmful combinatorial effects of a defective resolution of inflammation in the systemic circulation, aorta, and brain. For example, the function of neutrophils in both diseases leaves them as a promising target population for a more systemic approach to therapy, while the complex interplay between monocyte phagocytosis and release of inflammatory mediators in these two diseases may require a more nuanced approach that still allows for the clearance of oxLDL or Aβ while impeding the oncoming immune assault. With this in mind, the future of these two disparate diseases may not look as different as once thought.

## Author Contributions

All authors listed have made a substantial, direct, and intellectual contribution to the work and approved it for publication.

## Funding

The authors were supported by the National Institutes of Health under awards R01HL142129, R01HL139000, and HL142129-04S1 (EG).

## Conflict of Interest

The authors declare that the research was conducted in the absence of any commercial or financial relationships that could be construed as a potential conflict of interest.

## Publisher's Note

All claims expressed in this article are solely those of the authors and do not necessarily represent those of their affiliated organizations, or those of the publisher, the editors and the reviewers. Any product that may be evaluated in this article, or claim that may be made by its manufacturer, is not guaranteed or endorsed by the publisher.
